# Knowing and combating the enemy: a brief review on SARS-CoV-2 and computational approaches applied to the discovery of drug candidates

**DOI:** 10.1042/BSR20202616

**Published:** 2021-03-19

**Authors:** Mateus S.M. Serafim, Jadson C. Gertrudes, Débora M.A. Costa, Patricia R. Oliveira, Vinicius G. Maltarollo, Kathia M. Honorio

**Affiliations:** 1 Department of Microbiology, Biological Sciences Institute, Federal University of Minas Gerais (UFMG), Belo Horizonte, MG, Brazil; 2 Department of Computer Science, Federal University of Ouro Preto (UFOP), Ouro Preto, MG, Brazil; 3 Department of Pharmaceutical Products, Faculty of Pharmacy, Federal University of Minas Gerais (UFMG), Belo Horizonte, MG, Brazil; 4 School of Arts, Sciences and Humanities, University of São Paulo (USP), 03828-000, São Paulo, SP, Brazil; 5 Center for Natural and Human Sciences, Federal University of ABC (UFABC), Santo Andre, SP, Brazil

**Keywords:** Artificial intelligence, COVID-19, Drug discovery and design, Machine Learning, SARS-CoV-2

## Abstract

Since the emergence of the new severe acute respiratory syndrome-related coronavirus 2 (SARS-CoV-2) at the end of December 2019 in China, and with the urge of the coronavirus disease 2019 (COVID-19) pandemic, there have been huge efforts of many research teams and governmental institutions worldwide to mitigate the current scenario. Reaching more than 1,377,000 deaths in the world and still with a growing number of infections, SARS-CoV-2 remains a critical issue for global health and economic systems, with an urgency for available therapeutic options. In this scenario, as drug repurposing and discovery remains a challenge, computer-aided drug design (CADD) approaches, including machine learning (ML) techniques, can be useful tools to the design and discovery of novel potential antiviral inhibitors against SARS-CoV-2. In this work, we describe and review the current knowledge on this virus and the pandemic, the latest strategies and computational approaches applied to search for treatment options, as well as the challenges to overcome COVID-19.

## Introduction

In late December 2019 at Wuhan (China), an unknown acute respiratory disease was reported. At the first week of January 2020, a new virus called the severe acute respiratory syndrome-related coronavirus 2 (SARS-CoV-2) was identified as the etiological agent of the related cases, which would be later named as the coronavirus disease 2019 (COVID-19) [1,2]. Rapidly progressing from a local outbreak to a pandemic scenario, by the end of November 2020, the SARS-CoV-2 infection had been diagnosed in more than 57.8 million people, with almost 50% of cases in the Americas and over 11,789,000 of them in the United States of America (U.S.A.). Until 24 November 2020, over 1,377,000 deaths happened worldwide, with 252,460 (18.3%) in the U.S.A., due to the rapid SARS-CoV-2 spread and the severity of COVID-19 [2,3].

**Figure 1:**
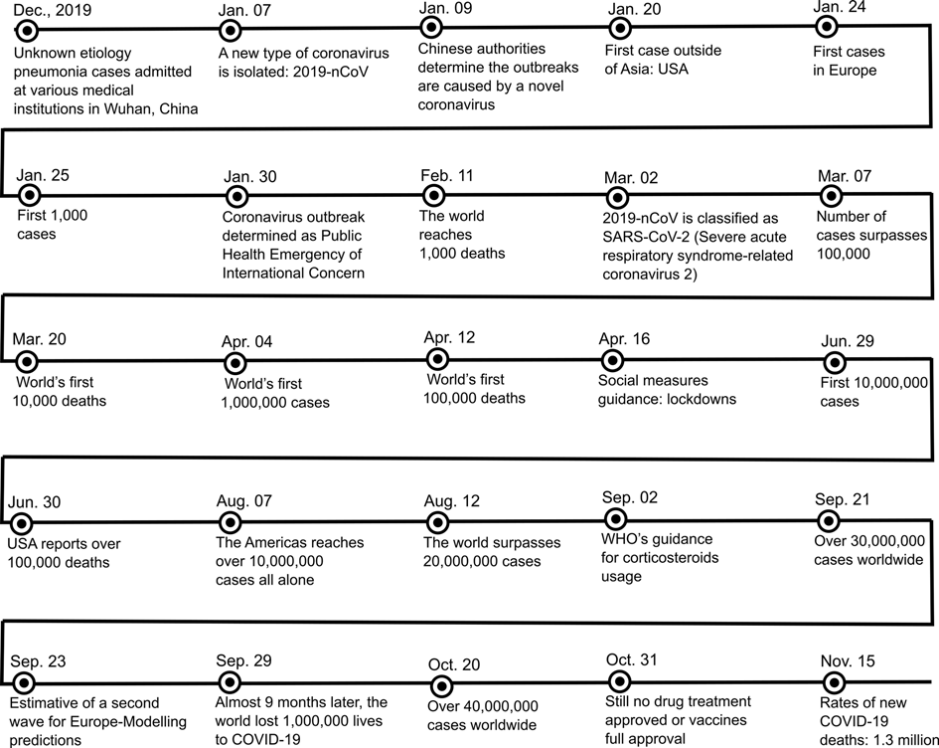
Main events related to SARS-CoV-2 during the year of 2020 (until 24 November)

Figure 1 presents an overview of the main events occurring during the year of 2020 and related to SARS-CoV-2, until the submission of this document. It comprises several key events, especially the number of deaths and the efforts made by the World Health Organization (WHO).

The coronaviruses, such as SARS-CoV-2, are from a large family (*Coronaviridae*) of spherical and enveloped virus, with 120–160 nm in diameter, and a single-stranded positive sense RNA (ssRNA+) genome, containing approximately 26–32 kb. These viruses were named due to the solar crown (‘corona’, in latin) aspect that the viral particles exhibit under electron microscopy due to the glycoproteins at its surface [4,5].

Transmission of coronaviruses usually occurs by air, oral–fecal routes or fomites, associated with gastrointestinal and respiratory infections, due to its tropism for epithelial cells [6–8]. Generally, these infections are asymptomatic and mild, but some species of coronavirus cause serious diseases, such as hepatitis, neurological disorders, kidney failure, and severe acute respiratory syndrome (SARS), which can lead to death [6,9].

There are six known human coronaviruses (HCoV) species, such as HCoV-229E and HCoV-NL63 (*Alphacoronavirus* genus), as well as HCoV-OC43 and HKU1 (*Betacoronavirus* genus), which mainly cause common colds, but can progress to severe infections of the lower respiratory tract, especially in children and the elderly. In addition to these betacoronaviruses, two other species are highly virulent: the Middle East respiratory syndrome coronavirus (MERS-CoV) and the severe acute respiratory syndrome-related coronavirus (such as SARS-CoV and SARS-CoV-2) [1,10,11].

The transmission of SARS-CoV-2 occurs through airways and from direct contact, in addition to the contact with contaminated objects and surfaces. Incubation periods may last up to 14 days (average of 5 days), and the most common symptoms are fever, cough, fatigue, headache, and breathing difficulties (dyspnoea), as well as smell and taste loss that can last over 2 weeks. In addition to these, sore throat, myalgia, diarrhea, vomiting, and nasal congestion can also occur [12–15].

Similar to other ssRNA+ viruses, after infection of host cells, progeny occurs in the cytoplasm. During its infection, SARS-CoV-2 particles bind to receptors at the cell surface by interaction of the receptor binding domain (RBD) of the spike protein (S) with the cell receptor, the angiotensin II converting enzyme (ACE-II) [4,10]. After the virus entry to the cells, during the expression of the viral replication complex, RNA is translated into two polyproteins (PP1a and PP1ab), which encode 16 non-structural proteins (NSPs), such as the main protease (M^pro^) and the RNA-dependent RNA polymerase (RdRp). The remainder of the genome encodes accessory and the structural proteins (spike, membrane (M), nucleocapsid (N), and envelope (E)), followed by the assembly and release of viral particles [4,16].

Regarding the infection control measures, vaccines are considered a viable and important alternative, in particular as the first line of prevention, even more in a pandemic scenario [17,18]. Some studies on different vaccine candidates to prevent SARS-CoV-2 infections have moved into Phases II and III trials, such as Pfizer-BioNTech [19], AstraZeneca-Oxford [20], Moderna [21], Gamaleya [22], as well as Sinovac [23], some of which have successfully completed Phase III clinical trials and/or licensed for early use in late November 2020. However, over the long-term efficacy, safety or global production in large-scale and short period of time may still be a challenge to overcome, and an immunization failure or insufficient coverage are not discarded, as observed with vaccines against other coronaviruses, such as SARS-CoV and MERS-CoV [16,24].

On the other hand, drug repurposing strategies could be promising in the fight against COVID-19. Approved or licensed drugs, as previously assessed by studies that evaluated them in preclinical and clinical trials, show that these approaches could potentially reduce time and costs for making new therapies available [25]. Considering this scenario and the possibility of novel outbreaks or pandemics, an approved drug may be also used to treat diseases caused by other coronaviruses or even future mutations of SARS-CoV-2 [26–28]. Different repurposing strategies and drug combinations have been proposed (e.g. remdesivir) but showed lack of inhibitory activity or inconclusive clinical results, as well as having to take the account of side effects, thus leaving a significant opportunity for the design and development of efficient drugs to face the challenges of SARS-CoV-2 and COVID-19 [29,30].

It is interesting to mention that *in silico* virtual screening approaches associated with structural and biophysical techniques can help the design of specific inhibitors to SARS-CoV-2, and significantly enhance the quality of compounds selected for *in vitro* and *in vivo* bioassays, increasing the success of drug discovery [31–34]. For instance, structure-based approaches have shown some successful outcomes in the past, for example, the design and discovery of boceprevir, an approved hepatitis C virus (HCV) protease inhibitor, as well as oseltamivir and zanamivir, both anti-influenza drugs [35].

In the past few months, several small molecules have been described as possible inhibitors of different molecular targets for SARS-CoV-2 [36]. However, it is important to note that many of these studies are still in the initial *in silico* analyses, which only provide a preliminary theoretical view on the ligand–protein interactions and hence requiring experimental validation of the molecular targets [33].

**Figure 2:**
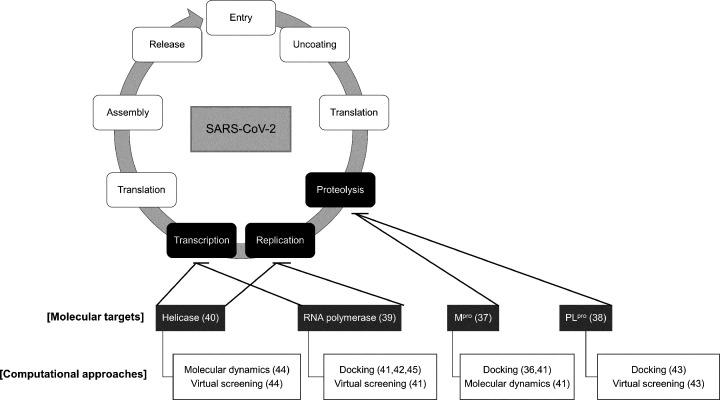
Interventions of computational approaches on to the design and discovery of potential inhibitors against molecular targets involved in the SARS-CoV-2 viral cycle The diagram displays available methods that can be employed to both the M^pro^ and PL^pro^ proteases (e.g. docking), as well as the RNA polymerase and helicase (e.g. virtual screening).

Among the molecular targets of SARS-CoV-2, main protease or 3-chymotrypsin-like protease (M^pro^/3CL^pro^/nsp5) [37], papain-like protease (PL^pro^/nsp3) [38], RNA-dependent RNA polymerase (RdRp/nsp12) [39], and helicase/NTPase (nsp13) [40] could be cited, which are highly conserved and essential to the viral cycle [36,41–45], as illustrated in Figure 2. Since the main viral protease is extensively studied for the design of new drug candidates to treat coronaviruses diseases, and most of the studies identified this enzyme as a valid target for broad spectrum inhibitors, we will next focus on the discussion of this macromolecule in more details [33,46,47].

## Computer-aided drug design strategies as useful tools against SARS-CoV-2 macromolecules: targeting M^pro^

Computer-Aided Drug Design (CADD) involves widely employed computational approaches to discover and/or design new bioactive compounds. As examples of CADD techniques we can cite molecular docking, molecular dynamics (MD) simulations, pharmacophore modeling, similarity analysis, quantitative structure–activity relationship (QSAR) analysis, and machine learning (ML) techniques [48–51] that will be discussed later.

**Figure 3:**
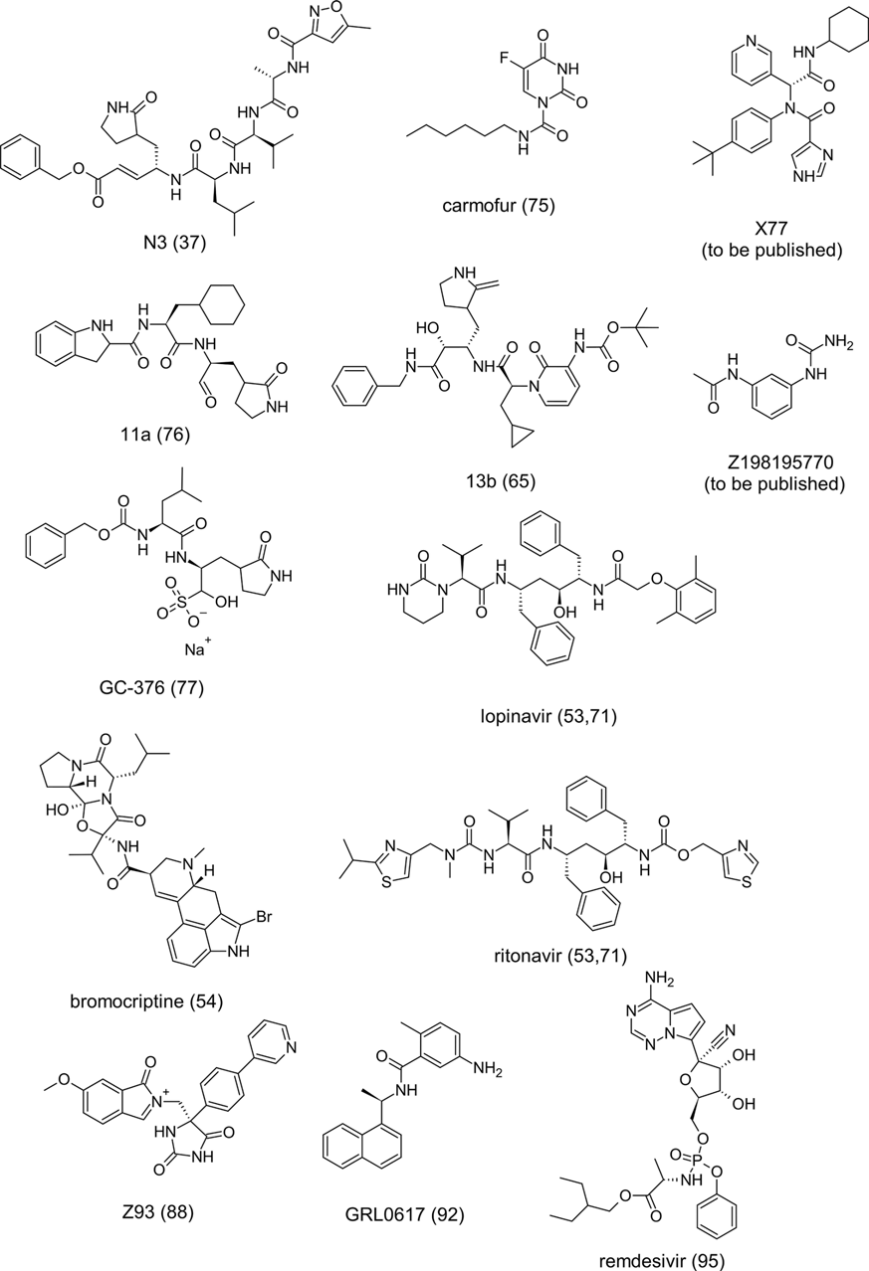
Two-dimensional (2D) chemical structures of the potential inhibitors and drug candidates presented in the present study, along with the respective references

Some recent studies have shown the feasibility of employing *in silico* methods such as molecular docking and MD simulations to perform virtual screening of molecules against the SARS-CoV-2 macromolecules and, consequently, obtaining potential repositioning drugs and selective inhibitors of these enzymes [52–54]. Figure 3 summarizes the molecular structures of the potential inhibitors and drug candidates discussed in this review.

M^pro^ (nsp5) is one of the most attractive viral targets for the antiviral drug discovery against SARS-CoV-2, since it plays a key role in the viral transcription and replication, and no human proteases are known with the same substrate specificity [37,46,47,55]. Furthermore, the substrate-binding pocket of this enzyme is highly conserved among all coronaviruses, suggesting that an antiviral drug targeting this active site may be effective against a broader spectrum of these viruses [37,56,57]. Nonetheless, mutations leading to changes in some amino acid residues of M^pro^ may provide probable drug resistance phenotypes, in particular considering the enzyme loop and the possibility of a protein folding [58]. In addition, protease resistance was also observed to MERS-CoV and other viruses, such as the human immunodeficiency virus (HIV) and HCV [59].

**Figure 4:**
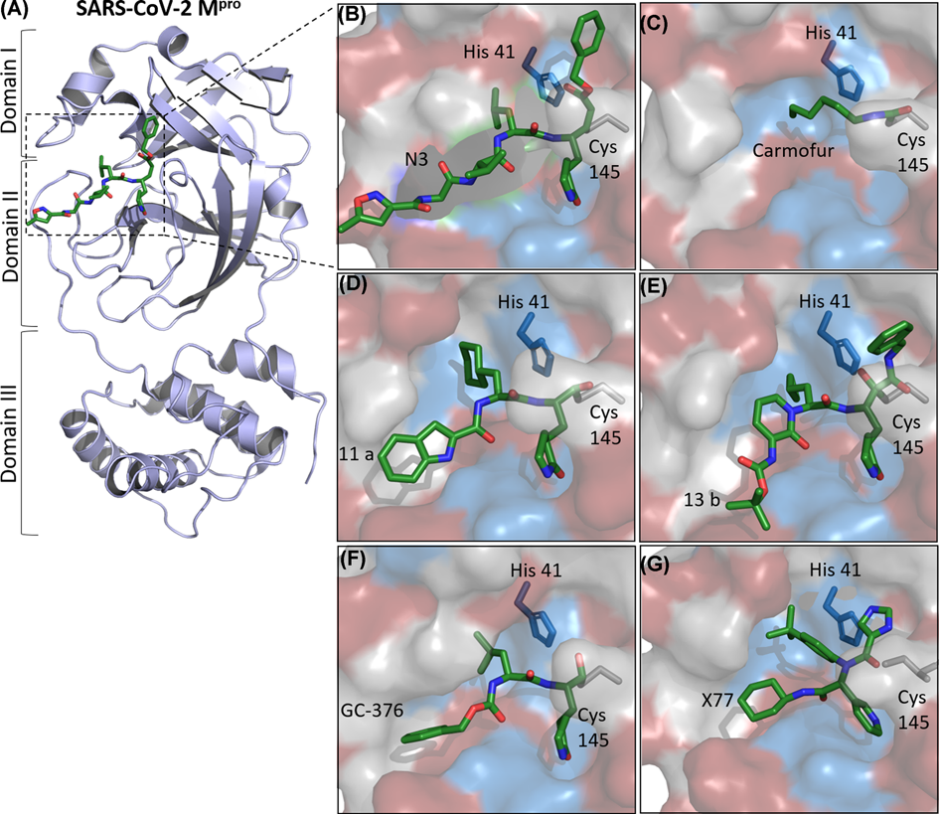
X-ray crystal structures of SARS-CoV-2 M^pro^ in complex with inhibitors (**A**) Cartoon representation of one protomer of the dimeric M^pro^ in complex with N3 (PDB ID: 6L7U). (**B–G**) An enlarged view of the substrate-binding pocket in a surface representation (red indicating hydrophobic residues, blue indicating charged residues, and gray indicating polar ones) in complex with different compounds. The catalytic Cys^145^ and His^41^ dyad are highlighted. (B) N3, PDB ID: 6LU7. (C) Carmofur, PDB ID: 7BUY. (D) 11a, PDB ID: 6LZE. (E) 13b, PDB ID: 6Y2F. (F) GC-376, PDB ID: 6WTT. (G) X77, PDB ID: 6W63. Images were generated with PyMOL 0.99 [68].

M^pro^ is a cysteine protease with a catalytic Cys^145^ and His^41^ dyad at its active site [60,61], which cleaves the polyproteins in at least 11 conserved sites, starting with its autolytic cleavage between nsp4 and nsp6 [62,63]. The M^pro^ structure is composed of three domains; the catalytic dyad is located in the cleft between domains I and II [37,64,65], and the domain III is responsible for the enzyme dimerization, enabling the active form of the macromolecule [66,67]. Figure 4 illustrates X-ray crystal structures of SARS-CoV-2 M^pro^ in complex with some inhibitors.

Several inhibitors of SARS-CoV M^pro^ have been identified [46,47]. For instance, N3 (Figure 4A,B), a Michael acceptor-based inhibitor, can specifically inhibit M^pro^ of different coronaviruses, including SARS-CoV and MERS-CoV [56,64,69,70]. It has also showed antiviral activity in cell culture against SARS-CoV-2 and the ability to bind to the substrate-binding pocket of SARS-CoV-2 M^pro^, as proven by X-ray crystallography (Figure 4A,B) [37].

Docking approaches can predict inhibitory activity and help drug design or virtual screenings, which have resulted in potential repurposing drugs against SARS-CoV-2 M^pro^ such as lopinavir and ritonavir [71]. The combination of docking and MD simulations, for example, allows a refinement of docking results, specifically evaluating the frequency of main interactions between residues and the drug candidates, as well as binding energy of the bioactive substances at the target, number of contacts, occupancy and stability of the target–ligand complex [72–74].

These strategies can also expand the number of compounds to be assessed in virtual screening (VS) campaigns, allowing thousands to millions of compounds to be screened from virtual chemical libraries. For instance, Jiménez-Alberto et al. (2020) showed bromocriptine, simeprevir and other FDA approved drugs to have promising inhibitory activity against the SARS-CoV-2 main protease, as result of VS and MD simulations [54]. Similarly, Kumar et al. (2020) also screened antivirals as drug repurposing therapies for COVID-19, and these studies highlighted lopinavir and ritonavir as potential treatments to be further evaluated in clinical trials against M^pro^ [53].

Another study investigated a combination of structure-based drug design, virtual and *in vitro* high-throughput screening of a library with more than 10,000 compounds, identifying disulfiram, carmofur, ebselen, shikonin, tideglusib, and PX-12 as SARS-CoV-2 M^pro^ inhibitors. Among these substances, ebselen exhibited the strongest antiviral effects in SARS-CoV-2-infected Vero cells in the low micromolar range [37]. The X-ray crystal structure of SARS-CoV-2 M^pro^ in complex with carmofur, an approved antineoplastic agent, also revealed that its carbonyl reactive group reacts irreversibly to bind to the catalytic Cys^145^ (Figure 4C) [75].

Moreover, some compounds designed and synthesized by analyzing the substrate-binding pocket of M^pro^ revealed anti-SARS-CoV-2 activity in Vero cell cultures. For example, the crystal structure of the complexes SARS-CoV-2 M^pro^-11a (Figure 4D) and M^pro^-11b indicated the presence of these substances inside the substrate-binding pocket and a similar inhibitory mechanism in which occurs the C–S covalent bond formation between Cys^145^ and these compounds [76].

Zhang et al. also developed an optimized α-ketamide inhibitor of SARS-CoV-2 M^pro^. The X-ray crystal structure of α-ketoamide (compound 13b) in complex with SARSCoV-2 M^pro^ shows the compound at the catalytic site of each protomer, between the domains I and II (Figure 4E). Compound 13b effectively prevented the viral replication in cell-based assays and exhibits a favorable pharmacokinetic *in vivo* profile [65]. Other work investigated boceprevir, GC-376, and calpain inhibitors effects on SARS-CoV-2 M^pro^ in enzymatic assays in cell cultures. The crystal structure of SARS-CoV-2 M^pro^ in complex with GC-376 also revealed molecular details on the GC-376 inhibition of the molecular target (Figure 4F) [77].

In addition to the studies above, several other structures of SARS-CoV-2 M^pro^ in complex with inhibitors have been deposited at the Protein Data Bank (PDB), such as a non-covalent inhibitor X77 (PDB ID: 6W63) (Figure 4G), narlaprevir (PDB ID: 7D10), boceprevir (PDB ID: 7COM), GRL-2420 (PDB ID: 7JKV) and UAW246 (PDB ID: 6XBG). However, most of these works have not so far been published in peer-reviewed papers.

Although additional research has also identified several inhibitors as promising drugs against SARS-COV-2 M^pro^, further biochemical and structural analyses, as well as *in vitro* and *in vivo* bioassays are still required. For example, several investigations predicted various small-molecules, natural compounds and approved drugs [78–80] from VS of ZINC and DrugBank databases [81,82], in conjunction with the combination of molecular docking and MD studies [53]. Focusing on drug repurposing, we can also highlight a study that apply molecular docking associated with the SCAR (steric-clashes alleviating receptors) protocol, which can help to discovery of covalent and non-covalent inhibitors in a docking model, solving, for example, steric conflicts between specific residues and reactive atoms in a screening [83]. In addition, it can even be efficient in drug repurposing [84], another approach of interest facing the urgency of the COVID-19 pandemic scenario.

It is important to mention that other strategies can evaluate and predict important characteristics of potential candidates and inhibitors of SARS-CoV-2 targets, for example, pharmacokinetics (absorption, distribution, metabolism, and excretion—ADME) and toxicity or simply ADME-Tox properties. Hage-Melim et al. (2020) screened for potential M^pro^ inhibitors and the top 100 hits were evaluated by bidimensional structural similarity in order to assess their ADME-Tox properties, resulting in ten compounds, including potential repurposing drug candidates such as lopinavir, ritonavir, and remdesivir [5]. Nonetheless, in a drug repurposing approach, this kind of evaluation may not be necessary, and a given candidate could proceed quickly to clinical trials, due to the previously assessed steps of an approved or licensed drug [85].

## The importance of multitargets to CADD approaches: PL^pro^ and RdRp

Another essential protease for the cleavage of the viral polyproteins is PL^pro^, a cysteine protease with a classical Cys-His-Asp catalytic triad (Cys^112^, His^273^, Asp^287^), which cleaves the viral polyprotein releasing nsp1, nsp2 and nsp3 [86,87]. This enzyme also recognizes the consensus cleavage sequence identified by cellular deubiquitinating enzymes [87]. Therefore, substrate-derived inhibitors of PL^pro^ would be expected to inhibit host cell deubiquitinases [41,76,87].

Computational approaches have also been used to predict potential SARS-CoV-2 PL^pro^ inhibitors. From integrated *in silico* efforts, Mirza et al. pointed out a human ubiquitin carboxyl-terminal hydrolase-2 (USP2) inhibitor (compound Z93) as a potential lead compound against SARS-CoV-2 PL^pro^ [88]. A pharmacophore model of functional centers of PL^pro^ inhibitor-binding pocket and docking studies also identified 147 FDA-approved drugs, including HIV, hepatitis C, and cytomegalovirus (CMV) inhibitors, as well as drugs that have demonstrated some activity in MERS and SARS-CoV as potential opportunities for the treatment of COVID-19 [89].

**Figure 5:**
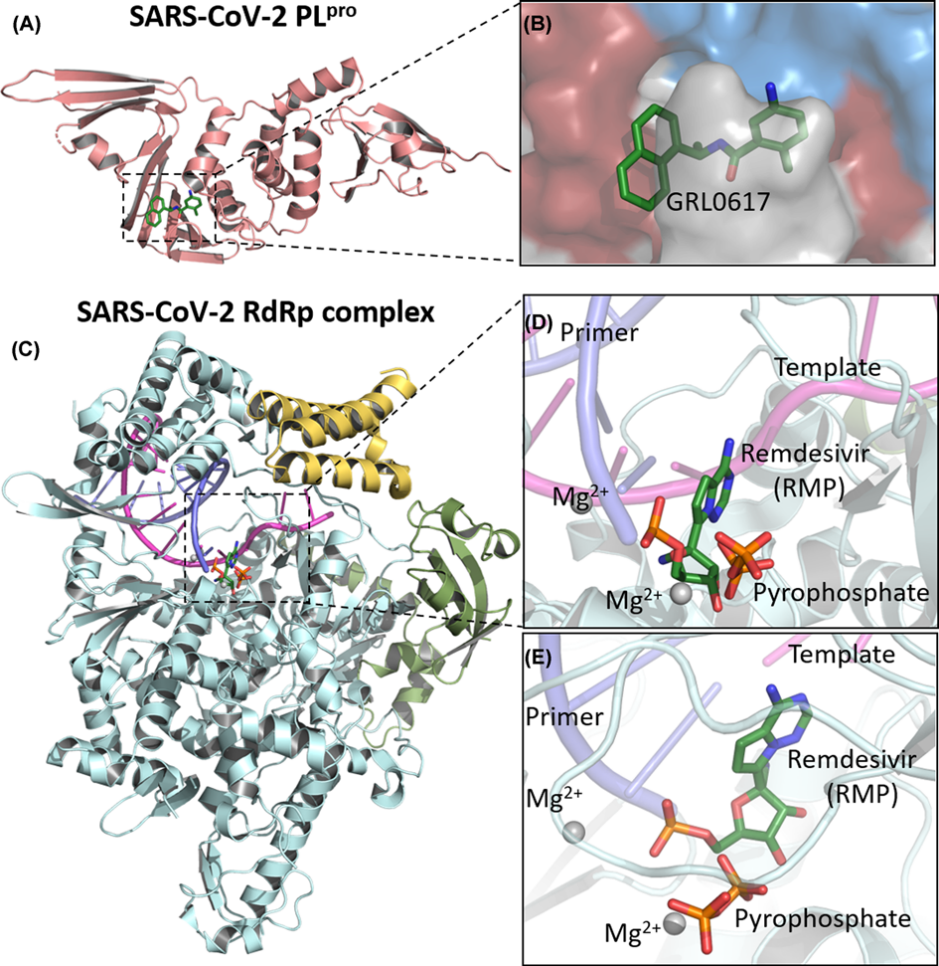
Crystal structure of SARS-CoV-2 PL^pro^ in complex with inhibitor GFL0617 and cryo-electron microscopy structure of the SARS-CoV-2 remdesivir and RNA bound RdRp complex (**A**) Cartoon representation of papain-like protease, PDB ID: 7CMD. (**B**) An enlarged view of PL^pro^ substrate-binding pocket with GFL0617. (**C**) Cartoon representation of the nsp12–nsp7–nsp8 RdRp complex with a template-primer RNA and remdesivir. The structure is colored by the elements: nsp12 in pale cyan, nsp7 in pale yellow, nsp8 in bright orange, primer RNA in purple and template RNA in light magenta, PDB ID: 7BV2. (**D**) Close-up view on the RdRp active site showing the covalently bound remdesivir in its monophosphate form—RMP, pyrophosphate and magnesium ions represented by gray spheres. (**E**) RdRp binding-pocket in a different view. Images were generated with PyMOL 0.99 [68].

Virtual screenings using ZINC and Chinese natural product databases [81], as well as FDA-approved drugs [90], have also found potential PL^pro^ inhibitors. Nonetheless, alternative approaches, such as assessing inhibitors activity in Vero cell cultures [91], as well as *in vitro* protease and structural assays [92], can also be mentioned. The crystal structure of GRL0617 in complex with SARS-CoV-2 PL^pro^ demonstrated that the inhibitor occupies the active site of the enzyme (Figure 5A,B) [92]. In addition, inhibition of the viral cycle has been demonstrated by GRL0617 against SARS-CoV [93].

The RdRp (nsp12) has also been described as an important coronavirus target for drug design [41,44,45]. It has an essential role in the viral cycle of coronaviruses, since it is responsible for the replication of the viral genome, with the assistance of nsp7 and nsp8 viral proteins in a polymerase complex [39,94,95]. In this sense, RdRp is considered as a primary target for nucleotide analog antiviral inhibitors such as remdesivir [96,97], which has been investigated in clinical trials against COVID-19. One study indicated that this drug seems to be capable of reducing the recovery time of severe hospitalized patients [98]. Regarding studies on the molecular target validation for this drug, the structure of the complex nsp12–nsp7–nsp8 associated with template-primer RNA and remdesivir shows that it is covalently incorporated at the first replicated base pair into the primer strand, blocking the RNA chain elongation (Figure 5C–E) [95].

Other studies applied a molecular docking approach and identified galidesivir, remdesivir, ribavirin, sofosbuvir, and tenofovir as potential drug candidates against SARS-CoV-2 RdRp [99]. Elfiky (2020) used sequence analysis, modeling and docking, identifying sofosbuvir, IDX-184, ribavirin, and remdesivir as potential therapies [100]. Beclabuvir, an HCV RdRp inhibitor, has also been predicted to bind SARS-CoV-2 RdRp [101].

Helicase/NTPase (nsp13) has also been cited as a molecular target of SARS-CoV-2. This macromolecule is responsible for unwinding DNA and RNA, separating them into two single-stranded nucleic acids in the coronaviruses viral cycle [102,103]. Molecular docking analyses and structure modeling approaches have also suggested drugs and natural products as potential SARS-CoV-2 helicase inhibitors [80,104].

In addition, a cryo electron microscopy (cryo-EM) structure of the complex nsp13:holo-RdRp:RNA complex suggests a possible role of helicase (nsp13) in the viral replication/transcription process, which needs to be confirmed through *in vitro* or *in vivo* studies [40]. Crystal structure of helicase in complex with Z198195770 (PDB ID: 5RL6), as well as other different compounds, has been determined, which can increase the recognition of this enzyme as a druggable target against SARS-CoV-2. However, *in vitro* and *in vivo* studies are necessary to confirm this macromolecule as an effective molecular target for the development of drugs against COVID-19.

## ML during a pandemic: actual state and future perspectives against SARS-CoV-2

**Figure 6:**
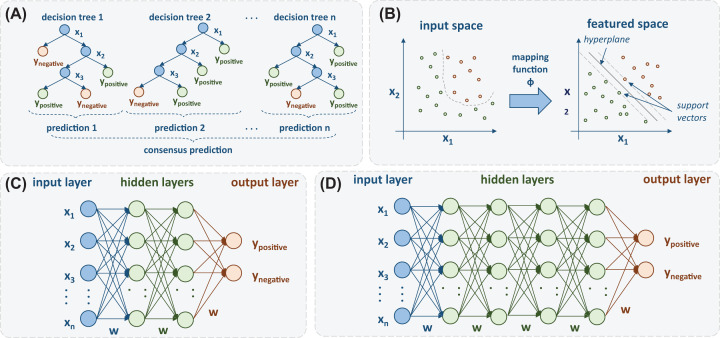
ML approaches (**A**) Random Forest (RF); (**B**) SVM; (**C**) Multilayer Perceptron (MLP) and (**D**) Deep Neural Network (DNN).

Machine learning (ML) techniques are a valuable new tool for drug discovery against SARS-CoV-2, since they can be applied to build predictive models based on previous experience (e.g. by using as training instances molecules already tested against other coronaviruses targets). ML applications in drug design commonly involves regression or classification methods for assessing the activity of compounds against a target before clinical trials. In this paper, we present the main aspects of ML approaches that may accelerate COVID-19 drug discovery, such as Ensemble Learning, Support Vector Machines (SVM), Artificial Neural Networks (ANN), and Deep Learning (DL) models [105–107]. In addition, integration of Artificial Intelligence (AI) and mechanistic modeling of signal transduction circuits, with ML algorithms, are also some recent approaches to drug repurposing models for COVID-19 [108,109]. Figure 6 presents an overview of some ML techniques.

Ensemble learning is a simple and effective learning model. The basic idea behind this approach is to build multiple models and combine their decisions in some manner [110]. Random forest (RF), illustrated in Figure 6A, is an ensemble of decision trees that uses the concept of bagging, which consists of randomly select samples to create the individual trees. Such random behavior leads to a more diverse set of decision trees, thus producing combined predictions that are more accurate than any of the individual ones. Similar approaches to RF include extra trees [111] and XGboost [112].

Aiming at discovering potential candidates to treat COVID-19, Rodrigues et al. (2020) applied computational tools to investigate the potential of diterpenes in inhibiting M^pro^ [113]. The authors collected a set of molecules from ChEMBL and Sistematx [114] databases to construct QSAR models using RF and multidescriptor read-across (MuDRA) models [115]. The models were used to perform a ligand-based VS, combined with a structure-based VS using Molegro Virtual Docker v6.0.1 [116]. Although no antiviral activity assays were performed, four diterpenes were selected as potential active inhibitors against the six different species of HCoV.

Similarly, Alves et al. (2020) presented a detailed study employing both structure and ligand-based computational approaches to select a set of compounds with the potential to inhibit M^pro^ [117]. In this work, RF was used to construct binary QSAR models with a set of molecules obtained from the ChEMBL and PBD databases. Different descriptors were used to build the RF models, resulting in a set of 42 potential hits from the DrugBank database, 11 of which were also available in the National Center for Advancing Translational Sciences (NCATS) pharmaceutical collection. The authors’ predictions found three compounds to be active and comparable to the cytopathic effect (CPE) assay data of NCATS.

Another important method used in QSAR studies is SVM (Figure 6B), which is a supervised approach based on the statistical learning theory [118]. The SVM algorithm incorporates the principles of structural risk minimization in its learning process and the reduction in complexity of mathematical functions used by the classifier. In summary, SVM constructs a separating hyperplane that maximizes the distance between the classifier and the nearest sample of each class, defined as margin separation [105]. Support vectors are the data points that lie closest to the decision surface (or hyperplane).

Although SVM presents an elegant mathematical formulation and good predictive performance, it also has some limitations, including the importance of making the right choice of hyperparameters and difficult interpretation of models. Fortunately, different strategies for tuning SVM hyperparameters can be found in literature [119,120].

Kowalewski and Ray (2020) developed a ML drug discovery pipeline to identify drug candidates for COVID-19. Initially, they collected assay data for 65 human targets with known interaction with SARS-CoV-2 proteins. SVM (for classification and regression) and RF were applied to predict the inhibitory activity and to screen FDA registered compounds and approved drugs. The predictions were filtered according to the estimated mammalian toxicity and vapor pressure with the aim at identifying volatile candidates and other inhibitors against multiple targets [121].

Inspired by the biological neural system, ANN are also interesting ML approaches. Multilayer perceptron (MLP) (Figure 6C) is a feed-forward neural network that has been widely applied in regression and classification tasks [110]. Its architecture consists of an input layer of neurons, an arbitrary number of hidden layers, and an output layer. Since the numbers of hidden layers and hidden neurons strongly influence the performance of the model, it is crucial to employ the right strategy to select hyperparameters in order to avoid overfitting and increase the generalization power of the network.

In comparison to MLP, DL neural networks architectures consist of a more complex set of hidden layers (Figure 6D) [122]. Such models contain hundreds to millions of units and require a great amount of training data to learn parameters, which is a computationally intensive process. One of the main advantages of DL models is their ability to handle complex data (i.e. text and images) by accomplishing automatic feature extraction from raw data, also called feature learning. Another frequently mentioned benefit is related to the scalability of such networks, referring to their ability to adjust the trade-offs between response time and accuracy [123].

**Figure 7:**
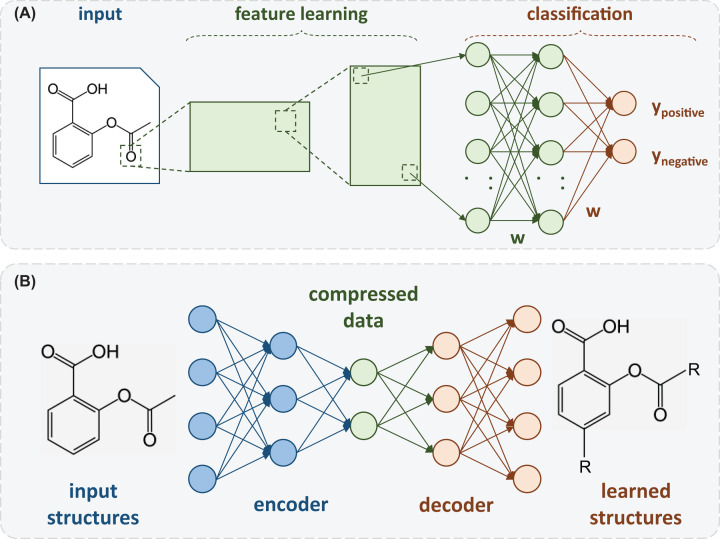
Special architectures of DL models (**A**) Convolutional Neural Networks (CNN) and (**B**) Autoencoders.

There are a number of DL architectures that carry out specialized tasks such as (i) convolutional neural networks, which perform object detection; (ii) recurrent neural networks, commonly used for time series analysis; and (iii) adversarial neural networks, that can learn about the input data and attempt to reconstruct it as faithful as possible by only using its underlying patterns [107]. In Figure 7, we present two DL architectures recently applied in studies involving SARS-CoV-2. Figure 7A presents an example of convolutional neural networks, while Figure 7B shows an example of adversarial neural networks.

DL models have also been applied in studies related to COVID-19 identification, starting from detecting some of the disease patterns in lung X-ray images [124]. Khandelwal et al. (2020) also selected a set of molecules as drug candidates to treat COVID-19 using DL. The authors undertook a careful study on 31 drug candidates against M^pro^ (PDB ID: 6LU7), where DL architectures were employed to generate shape-based molecules starting from a 3D shape of their seed compound’s pharmacophoric features. In this work, a convolutional neural network was trained as a variational autoencoder to learn the shape of PubChem’s chemical structures. Recurrent neural networks were applied to sample and generate new molecules that were evaluated according to physicochemical characterization and drug-likeness. Using this procedure, they obtained a set of molecules (remdesivir, valrubicin, aprepitant, fulvestrant, and a novel therapeutic compound named nCorv-EMBS) as potential inhibitors and possible therapeutics for COVID-19, after evaluation in antiviral activity assays [125].

Bung et al*.* (2020) combined a generative DL model, transfer learning, and reinforcement learning to design molecules capable of inhibiting M^pro^. The authors trained a generative model using a dataset with approximately 1.6 million drug-like molecules from ChEMBL. Transfer learning was employed to retrain the model with over 2,500 various protease inhibitor molecules, and reinforcement learning was applied to modulate the generative model aiming at producing molecules with desired properties. The trained model sampled 50,000 molecules from the chemical space, which were filtered based on many physicochemical properties. This process was followed by virtual screening and docking, resulting in a list of 31 compounds as potential hits which could be optimized into new therapies for COVID-19 [126]. Moreover, some important details of the docking methodology, such as definition of binding site, ligand and receptor preparation, and stereochemistry, have not also been described.

Gawriljuk et al. (2020) provided a detailed study of molecules as potential candidates against HeLa-ACE2. For this purpose, they compared different ML algorithms, including RF, support vector classification (SVC), k-nearest neighbors (kNN), and DL models, using a training dataset composed of 63 molecules and a test set containing 30 molecules. The results showed that RF and SVC presented the best prediction performance. It is worth mentioning that the DL models showed the poorest performance due to the lack of sufficient training data. The best models were employed in a virtual screening for selecting promising molecules, which were submitted to *in vitro* assays. Two of these compounds presented antiviral activity, with IC_50_ values of 8.4 μM and 540 nM, these representing useful potential starting points for COVID-19 focused drug discovery programs [106].

## Conclusions

Everyday, as more lives are lost, a common effort from science, government, and population tries to gradually tackle COVID-19, aiming to mitigate its continuously growing number of disease sequels, morbidities and deaths, with the urge and rapid progression of the current pandemic scenario. Taking into account that there are not approved or licensed drugs to treat COVID-19 so far, the race to find potential drug candidates benefits from computational strategies, which have proven to be a powerful tool, with the potential to obtain a successful combined approach in the arduous process of drug design and discovery. The urgency to obtain inhibitors and potential drug candidates remains as a major objective to mitigate the disease outcomes, including the death toll. CADD and ML techniques have been employed in many protocols targeting SARS-CoV-2 macromolecules and are one the feasible options to speed up the drug design and discovery processes, leading to novel inhibitors and repurposing drugs. In this review, we explored the potential of these many different approaches to find out or repurpose SARS-CoV-2 inhibitors as antivirals to treat COVID-19, facing, against the time, this pandemic scenario with an unflagging scientific effort.

## Abbreviations

ADME: absorption, distribution, metabolism, and excretion
ANN: artificial neural networks
CADD: computer-aided drug design
COVID-19: coronavirus disease 2019
DL: deep learning
MD: molecular dynamics
MERS-CoV: Middle East respiratory syndrome coronavirus
ML: machine learning
MLP: multilayer perceptron
M^pro^: main protease
NCATS: National Center for Advancing Translational Sciences
NSP: non-structural protein
PDB: Protein Data Bank
PL^pro^: papain-like protease
QSAR: quantitative structure–activity relationship
RdRp: RNA-dependent RNA polymerase
RF: random forest
SARS-CoV-2: severe acute respiratory syndrome-related coronavirus 2
ssRNA+: single-stranded positive sense RNA
SVC: support vector classification
SVM: support vector machine
